# Supplementary material for: KHSRP-mediated Decay of Axonally Localized Prenyl-Cdc42 mRNA Slows Nerve Regeneration
Source: bioRxiv. 2025 Feb 8:2025.02.06.636857. Preprint. [Version 1] doi: 10.1101/2025.02.06.636857 (PMC11839134; doi:10.1101/2025.02.06.636857)
Supplement: Supplement 1 — Supplemental Figure S1: Differential regulation of axonal Prenyl-Cdc42 and RhoA mRNA levels and translation (accompanying Figure 1–2). A) Representative IF images with no primary antibody as negative control for Figure 1D (see Figure 1E–F for quantifications). B-C) Representative FRAP image sequences for DRG neurons co-transfected with GFPMYR5’/3’Cdc42 (B), and mCherryMYR5’/3’RhoA (C) at 72 h post-transfection are shown. Boxed regions represent the photobleached ROIs (see quantification in Figure 2B–C) [Scale bar = 20 μm]. Supplemental Figure S2: Sequence alignment for vertebrate Prenyl-Cdc42 mRNA orthologs. Clustal Omega multiple sequence alignment (Sievers et al., 2011) for the 3’UTR of Prenyl-Cdc42 mRNAs are shown. Blue boxed regions show nucleotide conservation across orthologs. Nucleotide numbers labelled above start at the first nucleotide of the 3’UTR for Xenopus tropicalis. Beneath are graphical representations of consensus (% identity) and occupancy as well as a consensus aligned sequence. Supplemental Figure S3: Cell body expression of GFPMYR 3’prenyl-cdc42 mRNAs (accompanies data in Figure 3). A) Representative smFISH images for scramble FISH probe as negative control exposure matched to those in Figure 3B (see Figure 3D for quantitative data) [Scale bar = 10 μm]. B-C) Quantitation of smFISH signal intensities shown as mean pixel intensity above background ± SEM for cell bodies corresponding to Figures 3D–E (N ≥ 40 neurons in three independent cultures; NS = not significant between indicated data pairs by one-way ANOVA, pair-wise comparison with Tukey post-hoc tests). Supplemental Figure S4: KHSRP regulates axonal Prenyl-Cdc42 mRNA levels (accompanies data in Figures 4–5). A) Quantitation of smFISH signal intensities shown as mean pixel intensity above background ± SEM for cell bodies (see Figure 4D for representative images; N ≥ 30 neurons in three independent cultures; NS = not significant between indicated data pairs by one-way ANOVA with pair-wise com [file media-1.pdf]

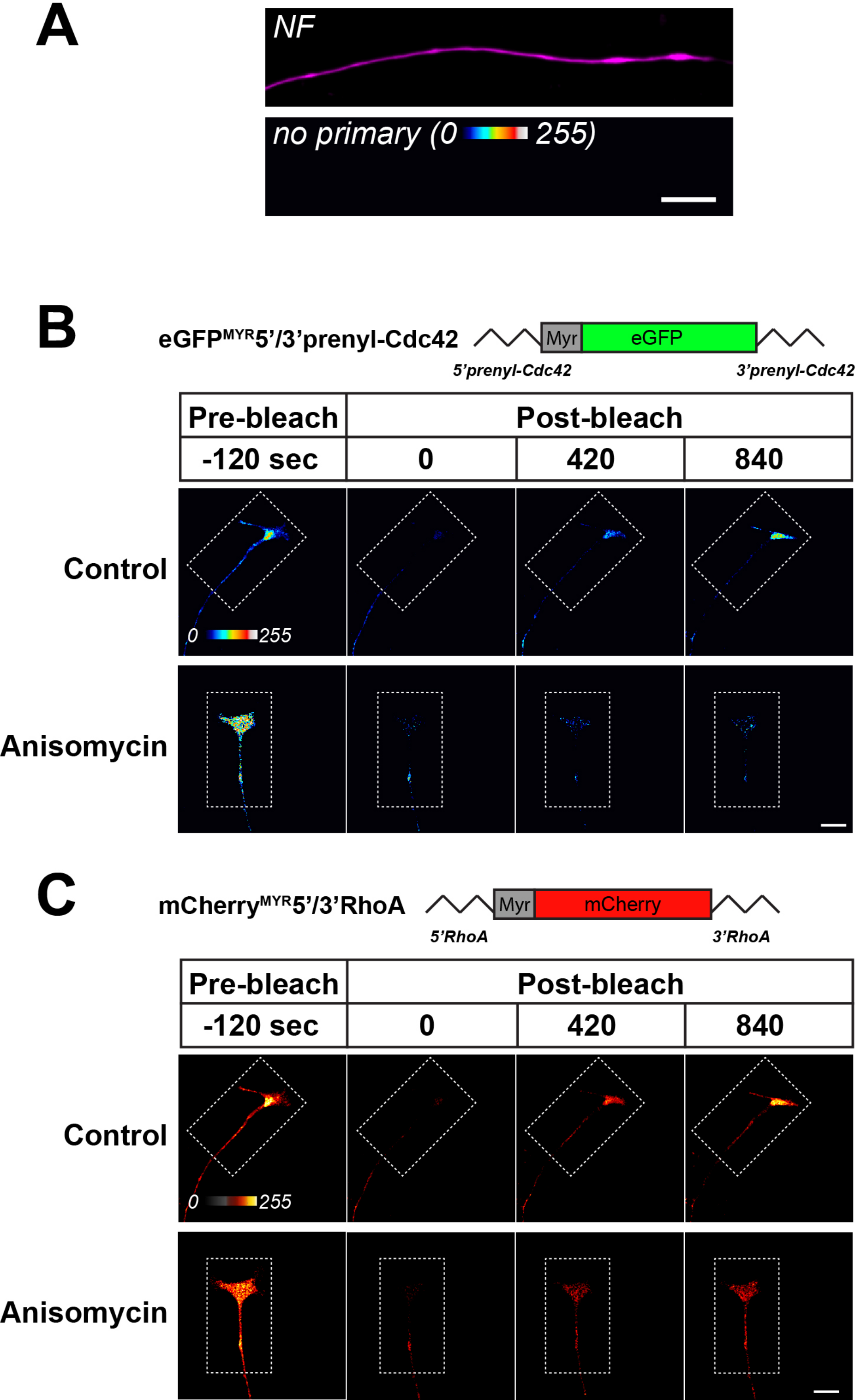
A
NF
no primary (0 255)
B
eGFPMYR5'/3'prenyl-Cdc42
Myr
eGFP
5'prenyl-Cdc42
3'prenyl-Cdc42
Pre-bleach
Post-bleach
-120 sec
0
420
840
Control
0
255
Anisomycin
C
mCherryMYR5'/3'RhoA
Myr
mCherry
5'RhoA
3'RhoA
Pre-bleach
Post-bleach
-120 sec
0
420
840
Control
0
255
Anisomycin

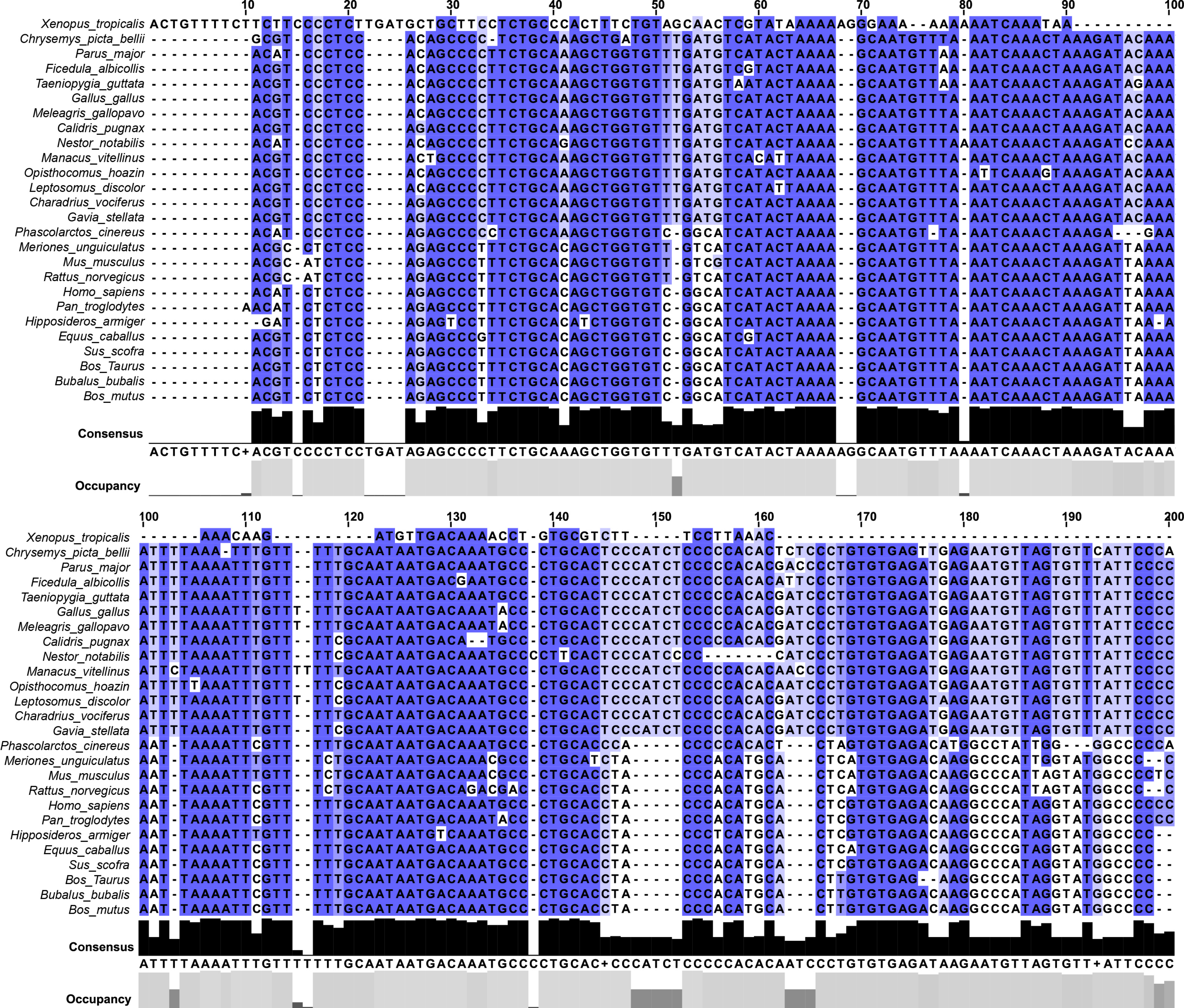
Xenopus_tropicalis
Chrysemys_picta_bellii
Parus_major
Ficedula_albicollis
Taeniopygia_guttata
Gallus_gallus
Meleagris_gallopavo
Calidris_pugnax
Nestor_notabilis
Manacus_vitellinus
Opisthocomus_hoazin
Leptosomus_discolor
Charadrius_vociferus
Gavia_stellata
Phascolarctos_cinereus
Meriones_unguiculatus
Mus_musculus
Rattus_norvegicus
Homo_sapiens
Pan_troglodytes
Hipposideros_armiger
Equus_caballus
Sus_scrofa
Bos_Taurus
Bubalus_bubalis
Bos_mutus
Consensus
ACTGTTTTC+ACGTCCCCTCCTGATAGAGCCCCTTCTGCAAAGCTGGTGTTTGATGTCATACTAAAAGGCAATGTTTAAAATCAAACTAAAGATACAAA
Occupancy
Consensus
ATTTTAAAATTTGTTTTTTTGCAATAATGACAAATGCCCCTGCAC+CCCATCTCCCCCACACAATCCCTGTGTGAGATAAGAATGTTAGTGTT+ATTCCCC
Occupancy

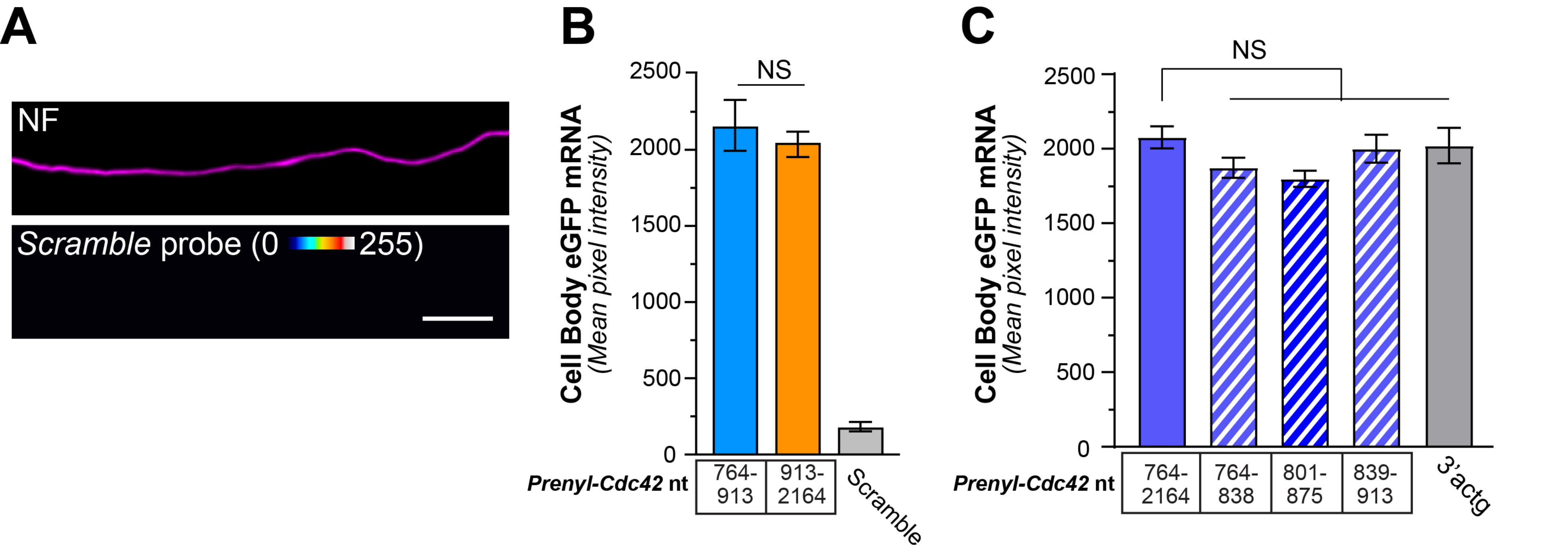

A
NF
Scramble probe (0 255)
B
NS
Cell Body eGFP mRNA
(Mean pixel intensity)
2500
2000
1500
1000
500
0
Prenyl-Cdc42 nt
764-913
913-2164
Scramble
C
NS
Cell Body eGFP mRNA
(Mean pixel intensity)
2500
2000
1500
1000
500
0
Prenyl-Cdc42 nt
764-2164
764-838
801-875
839-913
3'actg

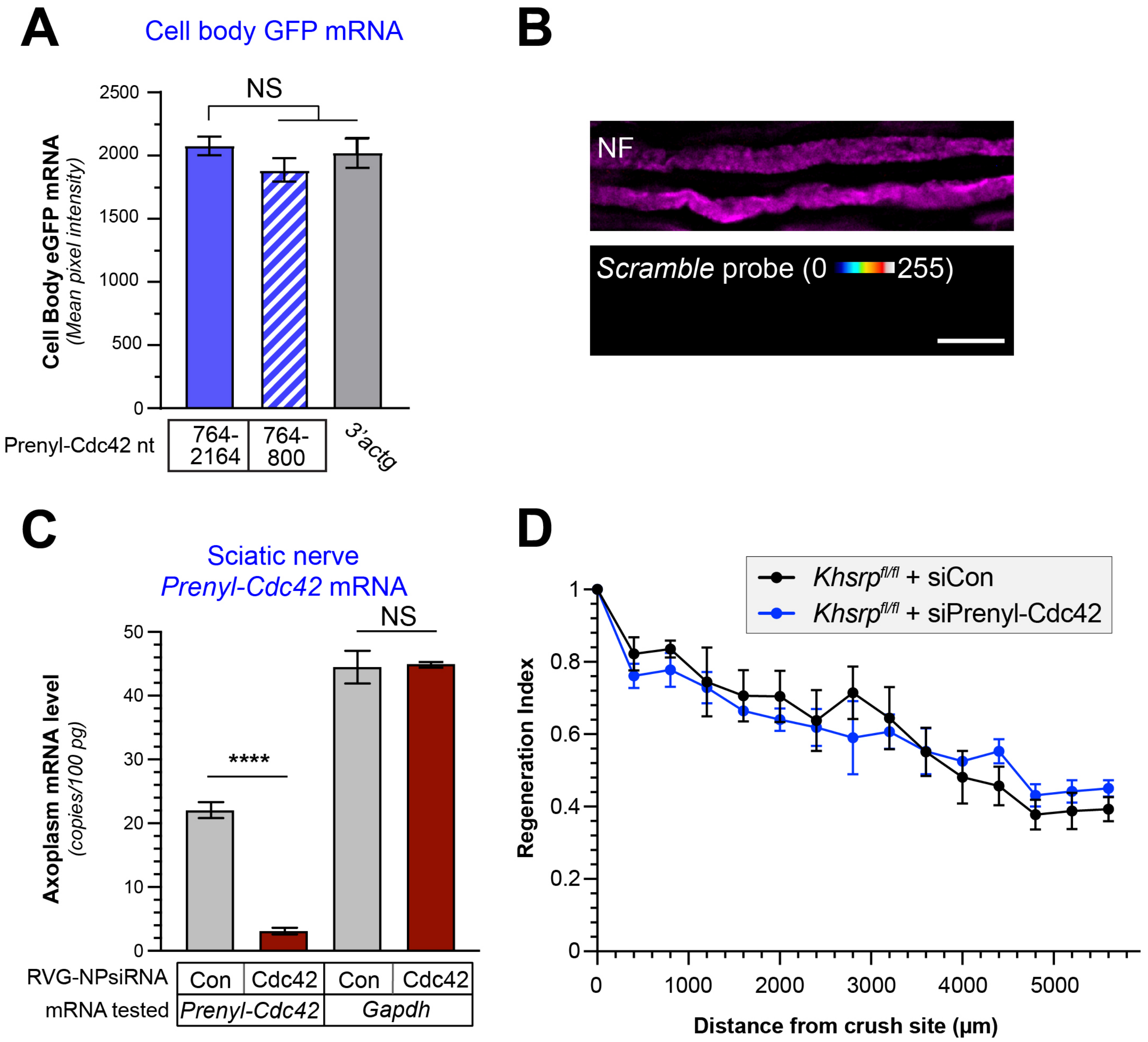

A
Cell body GFP mRNA
NS
Cell Body eGFP mRNA
(Mean pixel intensity)
2500
2000
1500
1000
500
0
Prenyl-Cdc42 nt
764-2164
764-800
3'actg
B
NF
Scramble probe (0 255)
C
Sciatic nerve
Prenyl-Cdc42 mRNA
Axoplasm mRNA level
(copies/100 pg)
50
40
30
20
10
0
****
NS
RVG-NPsiRNA
Con
Cdc42
Con
Cdc42
mRNA tested
Prenyl-Cdc42
Gapdh
D
Khsrp fl/fl + siCon
Khsrp fl/fl + siPrenyl-Cdc42
Regeneration Index
1
0.8
0.6
0.4
0.2
0
0
1000
2000
3000
4000
5000
Distance from crush site (μm)

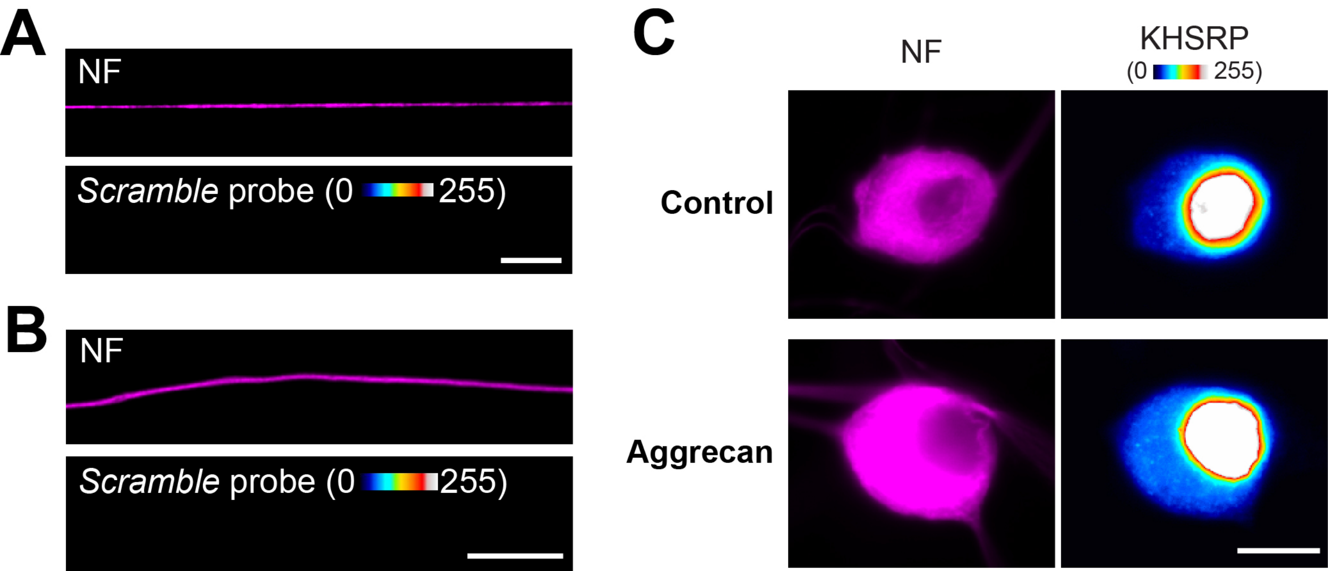
A
NF
Scramble probe (0 255)
B
NF
Scramble probe (0 255)
C
NF
KHSRP
(0 255)
Control
Aggrecan
